# Pain in the neurodegenerating brain: insights into pharmacotherapy for Alzheimer disease and Parkinson disease

**DOI:** 10.1097/j.pain.0000000000002111

**Published:** 2020-11-24

**Authors:** Timothy Lawn, Yahyah Aman, Katarina Rukavina, George Sideris-Lampretsas, Matthew Howard, Clive Ballard, Kallol Ray Chaudhuri, Marzia Malcangio

**Affiliations:** a Centre for Neuroimaging Sciences, The Institute of Psychiatry, Psychology, and Neuroscience, King's College London, London, United Kingdom; b Department of Clinical Molecular Biology, University of Oslo and Akershus University Hospital, Lørenskog, Norway; c The Maurice Wohl Clinical Neuroscience Institute, The Institute of Psychiatry, Psychology, and Neuroscience, King's College London, London, United Kingdom; d Parkinson Foundation Centre of Excellence, King's College Hospital, London, United Kingdom; e Wolfson Centre for Age Related Diseases, The Institute of Psychiatry, Psychology, and Neuroscience, King's College London, London, United Kingdom; f University of Exeter, Exeter, United Kingdom

## 1. Introduction

**Figure 1.**
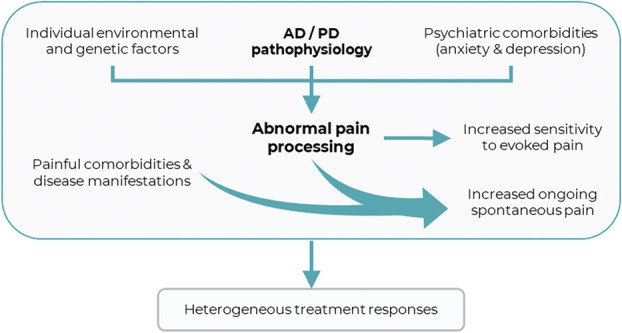
Conceptual framework relating the respective neurodegenerative pathophysiology within AD and PD to pain processing and treatment. AD, Alzheimer disease; PD, Parkinson disease.

The effects of neurodegeneration on the experience of pain remain poorly understood, despite the risk of suffering from both pain and neurodegenerative diseases rising concurrently with age.^63,75^ Given the anticipated increase in magnitude and median age of the global population,^76,152^ the interaction of these 2 clinically unmet needs will become an increasingly pressing challenge. In particular, a significant proportion of patients with Alzheimer disease (AD) and Parkinson disease (PD), the 2 most prevalent neurodegenerative diseases, suffer chronic pain of variable origin (Box 1). As such, they have been the most extensively studied and, for brevity, will be the focus of this review. Persistent pain in AD and PD is partially attributable to various concomitant disease manifestations and comorbidities (Fig. 1).^43,117^ In addition, disease-specific neurodegenerative changes may affect a multitude of regions implicated in the perceptual and cognitive processes underlying pain. Despite this, the precise perceptual sequelae of neurodegenerative pathophysiology in these 2 diseases remain equivocal, and whether this may result in differential responses to analgesic treatment remains largely unexplored.

Box 1. **Definitions**

Neurodegenerative disease: a heterogeneous group of disorders that are characterized by the progressive degeneration of the structure and function of the central nervous system or peripheral nervous system.

Dementia: a syndrome that involves severe loss of cognitive abilities as a result of disease or injury. Dementia caused by traumatic brain injury is often static, whereas dementia caused by neurodegenerative disorders, such as AD, is usually progressive and can eventually be fatal.

Alzheimer disease: a progressive neurodegenerative disease that impairs memory and cognitive judgment and is often accompanied by mood swings, disorientation, and eventually delirium. The most common cause of dementia.

Parkinson disease: a progressive neurodegenerative disorder, characterized by motor symptoms, such as tremor, rigidity, slowness of movement, and problems with gait. Motor symptoms are often accompanied with fatigue, depression, pain, and cognitive problems.

Three key principles lay conceptual foundations for the investigation of the effects of neurodegenerative pathophysiology on treatment mechanisms: (1) a given intensity of stimulus produces heterogeneous levels of reported pain and unpleasantness,^30,60,110,109,111,154^ (2) genetic and environmental factors predispose some to chronic pain,^1,47,48,87^ and (3) diversity of pain physiology and pathophysiology results in heterogeneous responses to pharmacotherapy^46,100,134,153^. Collectively, these support the notion that heterogeneous physiology and pathophysiology can give rise to divergent treatment responses. Within this framework, neurodegeneration and its effects on the central nervous system can be considered as one such external factor contributing to heterogeneity, resulting in putative perturbation of pain processing (1 and 2) and responses to analgesic treatments (3) (Fig. 1).

**Figure 2.**
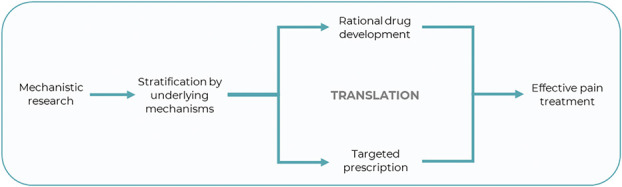
Importance of mechanistic research for evidence-based pain medicine.

Chronic pain in AD and PD not only impacts patients' quality of life but also presents a formidable healthcare and socioeconomic challenge. Drugs available for treatment of chronic pain are associated with high numbers needed to treat and may have serious side effects.^145^ Moreover, poorly managed pain is associated with depression,^33^ anxiety,^139^ and functional loss.^38^ Given the high prevalence of pain and frailty in these patient groups, clear scientific rationale is imperative to ensure safe and effective clinical management (Fig. 2). In this article, we discuss pain processing and perception in AD and PD as well as its emerging relevance to pharmacological treatment.

## 2. Alzheimer disease

Alzheimer disease is the most common form of dementia affecting more than 45 million people worldwide^119^ and is clinically characterised by progressive cognitive deterioration.^25,43,77^ The prevalence of chronic pain in dementia is between 30% and 80%.^43^ However, patients with AD do not report pain as often and are prescribed analgesics less frequently, compared with healthy age-matched individuals.^34,129^ Pain is a key trigger for behavioural and psychological symptoms of dementia such as agitation and mood disorders, which are a major treatment challenge and can result in overprescribing of harmful antipsychotic medications.^10,52,123^ Pathologically, the basal forebrain and medial temporal lobe are amongst the first regions affected before progression to neocortical regions.^18,108^ Notably, the sensory cortices remain relatively unaffected until terminal stages. The significance of this is multifaceted: (1) the regions affected partially overlap with regions implicated in the processing of pain, (2) the regions affected are believed to be involved more in emotional-affective rather than sensory-discriminative dimensions, and (3) the cognitive deficits within memory, attention, and communication render self-report of pain increasingly unreliable with disease severity. Specifically, a reduced capacity to comprehend and complete standardised pain assessments as well as an overall reduction in reporting of pain.^2,78,84,113^ Therefore, altered pain processing (1 and 2) is challenging to disentangle from a diminished capacity to accurately provide self-report (3), highlighting the need for investigation at a mechanistic level.

### 2.1. Pain processing is altered in Alzheimer disease

Many psychophysical studies investigating noxious stimuli have demonstrated altered pain processing in AD compared with healthy controls. However, the directionality of these changes remain equivocal. Thresholds have been reported to be increased^15,35,66,106^ or similar to cognitively intact controls.^15,81,79,82,93^ Similarly, pain tolerance has been reported to be reduced,^11,35,79,82^ equal,^35,66,81,89,88^ and increased.^122^ In addition, behavioural responses to pain have been shown to be augmented in AD,^72,89,88^ with enhanced facial responses throughout the spectrum of disease severity.^12^ Patients with AD have also shown a reduced threshold in the nociceptive flexion reflex (NFR), possibly indicating differences in pain processing further down the neuroaxis.^89^ Overall, disparities are likely due to differences in pathophysiological mechanisms, disease progression, modalities of evoked pain used, and, crucially, outcome measures used. Collectively, these findings allude to patients with AD potentially suffering more despite reporting pain less.

Neuroimaging studies have suggested that neural activity in patients with AD may be augmented in response to noxious stimulation, despite relative preservation of sensory-discriminative facets of pain. Patients show greater amplitude and duration of blood oxygenation level dependent (BOLD) signals (an indirect index of brain activity relating to neurovascular coupling) during noxious pressure stimulation within sensory, affective, and cognitive regions, including the dorsolateral prefrontal cortex (dlPFC).^35^ Consistent with altered cognition being functionally related to pain processing, patients also show enhanced functional connectivity between the dlPFC and anterior midcingulate, periaqueductal grey (PAG), thalamus, and hypothalamus.^36^ Indeed, the dlPFC plays a central role in both general cognitive function^70^ as well as pain modulation.^95,130,149^ Furthermore, diffusion tensor imaging has evidenced anatomical connectivity between the right dlPFC, hypothalamus, and PAG,^71^ in which activity has been associated with pain-related escape responses in rodents.^86,98^ This may reflect a failure to adequately contextualise and appraise painful experiences resulting in uncertainty and a higher threat value ascribed to noxious stimulation. Furthermore, a lack of contextualising features within scanning environments may compound this.^36^ Delineation of the impact of context and setting warrants further investigation. Collectively, neuroimaging studies indicate greater emotional reactivity and pain processing, despite equal or mildly diminished thresholds.

The implication of regions including the dlPFC, PAG, and hypothalamus overlaps with the neural substrates of placebo analgesia through which context and expectation can profoundly alter treatment responses.^36,118,150^ Patients with AD with reduced frontal lobe function exhibited diminished placebo responses in an open-hidden paradigm, requiring escalation of analgesic dose.^16^ Furthermore, executive function is the domain of cognition that best predicts variance in facial responsiveness to noxious electrical stimulation and the NFR.^90^ Thus, patients with milder disease severity may benefit more from analgesics because of relative preservation of placebo mechanisms. The placebo response is engaged in the administration of all pharmacotherapy to some extent and accounts for a large portion of the reduction in pain produced, over and above pharmacological efficacy.^14,17,37,148^ Therefore, patients with attenuated placebo responses should require larger doses to produce the same level of analgesia as controls. Worryingly, as AD and age progress, patients become increasingly frail, hence dose escalation may be a major concern given that age is a significant predictor of opioid-related harm.^28,57,85^ Placebo analgesia and opioid analgesia partially share neuroanatomical substrates; covariation has been observed between the activity in the rostral anterior cingulate cortex (ACC) and the brainstem during both placebo and opioid analgesia, but not during pain alone.^114,135^ Postmortem AD brains also show reduced μ-/δ-opioid receptor binding.^104^ Patients with AD may thus present alterations in centrally mediated opioid analgesia. Further application of open-hidden paradigms alongside pharmacoimaging may offer insights into how the combined magnitude of pharmacological and placebo analgesia can be maximised clinically.

### 2.2. Pharmacotherapy of pain in Alzheimer disease

Overall, patients with AD seem to be prescribed fewer analgesics than healthy individuals.^10,73,128^ Conversely, recent studies from Scandinavia have reported an opposite trend.^80,96,126^ Paracetamol/acetaminophen remain the principal treatment for mild-to-moderate pain in AD with additional use of nonsteroidal anti-inflammatory drugs and opioids.^3^ However, studies providing mechanistic insight remain scarce.^3,53^ For example, of the 3 randomised control trials (RCTs) investigating opioids, 2 were underpowered and in one investigating the buprenorphine transdermal system, 23 of the 44 patients withdrew treatment because of adverse events.^52,97,101^ No trials have investigated antidepressants and antiepileptics.^3,77^ Further RCTs will be necessary to not only produce evidence-based treatment guidelines but also to provide insights into the putative perturbation of neurotransmitter systems.

## 3. Parkinson disease

Pain is a prevalent nonmotor symptom in people with PD (PwP), acknowledged by James Parkinson in 1817,^112^ affecting 68% to 85% of patients.^13,23,103,116,127^ Despite this, it remains underdiagnosed and undertreated.^6,31,41,58,83,156^ Pain in PwP is multifaceted and may result from comorbidities, be caused or amplified by motor symptoms, and is subject to abnormal nociceptive processing, as PD-specific neurodegeneration affects peripheral, spinal, and cerebral pain pathways.^42,125^ Attempts have been made to synthesize a clear picture of heterogeneous pain in PD (Table 1)^6,58,151^; however, to date, our basic understanding of the relationship between PD pathophysiology and pain remains underdeveloped. Identifying well-defined subtypes, and elucidating their concomitant underlying mechanisms, should facilitate the development of personalised treatment of pain in PwP.^24,143^

**Table 1:** Overview of the classification systems to date for pain in people with Parkinson disease.

Quinn et al.^121^	A) Pain preceding diagnosis of Parkinson disease B) Off-period pain (without dystonia) in patients with a fluctuating response to levodopa 1. Morning pain 2. Wearing-off pain 3. Beginning-of-dose pain 4. End-of-dose pain C) Painful dystonic spasms 1. Early morning dystonia 2. Off-period dystonia 3. Beginning-of-dose dystonia 4. End-of-dose dystonia D) Peak-dose pain
Ford^61^	1. Musculoskeletal (aching, cramping, arthralgic, and myalgic sensations in joints and muscles) 2. Radicular/neuropathic (pain in a root or nerve territory) 3. Dystonia (associated with sustained twisting movements and postures) 4. Central or primary pain (burning, tingling, formication, and “neuropathic” sensations, often relentless and bizarre in quality) 5. Akathisia (subjective sense of restlessness, often accompanied by an urge to move)
Wasner and deuschl^151^	A) Pain related to Parkinson disease: **1. Nociceptive: Musculoskeletal** (joint pain, pain linked to motor fluctuations—dystonic or nondystonic, back pain, and pain linked to autonomic failure), **visceral** (abdominal pain, gastrointestinal discomfort, constipation, and involuntary dystonic contraction of anal sphincter), and **cutaneous** (pressure sores) **2. Neuropathic: Peripheral** (radicular) or **central Parkinson pain** **3. Miscellaneous:** pain preceding Parkinson disease, pain linked to restless leg syndrome and akathisia, and pain linked to depression. B) Pain unrelated to Parkinson disease —different pain syndromes.
Chaudhuri et al.^32^	1. Musculoskeletal pain (pain around joints) 2. Chronic pain (a generalised constant, dull, aching pain or pain related to an internal organ) 3. Fluctuation-related pain (dyskinetic pain, “off”-period dystonia, and generalised “off”-period pain) 4. Nocturnal pain (pain related to periodic limb movement and restless leg syndrome or pain related to difficulties turning around in bed) 5. Oro-facial pain (pain when chewing, pain due to grinding the teeth, and burning mouth syndrome) 6. Discolouration/oedema and swelling (burning pain in limbs and generalised lower abdominal pain) 7. Radicular pain (a shooting pain/pins and needles down the limbs)
Mylius et al.^107^	A) Non–Parkinson disease-related pain B) Parkinson disease-related pain: 1. Musculoskeletal pain 2. Psychomotor restlessness pain 3. Neuropathic pain

### 3.1. Pain processing is altered in Parkinson disease

Studies have largely reported reduced pain thresholds (greater sensitivity to pain) and lower pain tolerance in PwP (for meta-analysis, see Ref. 141). Interestingly, no relationship between pain sensitivity and disease duration was reported across 26 studies.^141^ Moreover, significant heterogeneity is seen within and across studies suggesting considerable interindividual differences with multiple contributory factors. Surveys have found intensity and frequency of pain to be higher in patients with more advanced PD; however, this likely reflects an increased incidence of musculoskeletal pain.^141^ A study using quantitative sensory testing failed to find a difference between drug-naive pain-free patients and controls suggesting that abnormalities may arise later in the disease duration, relate to dopaminergic therapy, or be associated with the development of chronic pain.^62^ In the absence of longitudinal investigation, the effects of disease progression are impossible to delineate but the power advantages of meta-analysis add credence to the possibility that enhanced pain sensitivity is engaged at a certain point during pathogenesis with a strong ceiling effect. Early pathophysiology within the midbrain and brainstem regions may therefore be important for elevated psychophysical pain sensitivity and reduced pain thresholds. Conversely, conditioned pain modulation paradigms, which assess the functionality of descending modulatory mechanisms, have been found to be comparable in controls and patients with PD in both ON and OFF states.^68,69^ However, trend significant differences were seen between PD subtypes (akinetic rigid, tremor dominant, and mixed). Given the low power of the study, this supports the heterogeneity of pain processing in PwP and emphasises the need for large studies that allow for adequately powered substratification.

Functional magnetic resonance imaging has revealed maladaptation of pain networks present even at early disease stages in pain-free PwP compared with healthy controls. Increased pain-related BOLD activation was observed in the somatosensory cortex, cerebellum, and caudal pons.^138^ Furthermore, activity in descending pain modulatory regions, such as the dlPFC, dorsal ACC, and subgenual ACC, is lower in PwP than in healthy individuals, and connectivity between dorsal ACC and dlPFC during anticipation of pain is reduced.^138^ The bilateral activation of the nucleus accumbens (NA) in PwP is also lower than that in healthy controls, suggesting altered processing of cognitive and evaluative facets of pain.^120,140^ A network-based analysis has shown dysfunction in reward pathways in PwP suffering from persistent pain, but not those without, with disconnection of the right NA and left hippocampus.^118^ The NA has been implicated in the transition from acute to chronic pain across a variety of human and animal studies.^8,29,51,56,155^ The direction of causality remains unclear, but dysfunction of reward and modulatory networks may predispose PwP to develop chronic pain and offer therapeutic targets.

### 3.2. Pharmacotherapy of pain in Parkinson disease

Pain in PwP remains neglected and poorly understood, with only a minority of patients receiving adequate treatment.^13^ People with PD are more likely to be prescribed analgesics, such as opiates, acetaminophen, antiepileptics, and antidepressants, as well as receive chronic prescriptions, risking polypharmacy or burdensome side effects.^22^ Dopaminergic replacement therapy might lead to pain relief in some PwP.^92,142^ For example, a 2-fold improvement in the King's Parkinson Disease Pain Scale domain “fluctuation-related pain” was observed with rotigotine vs placebo.^124^ l-Dopa administration reversed the reduction of pain threshold seen in PwP during the off-state^64^ and normalised abnormally increased pain-related activation within sensory-discriminative (insula) and cognitive-affective (prefrontal cortex and ACC) regions in a positron emission tomography study.^21^ Interestingly, pain reduction from l-dopa administration or deep brain stimulation [for review, see; Refs. 39,45,91] does not correlate with motor improvement suggesting it may act directly on pain circuitry.^40,92,102,142^ l-Dopa is not only converted exclusively into dopamine but also into noradrenaline and may act as a false neurotransmitter within serotonergic terminals.^50^ As both monoamines play a role in descending pain modulation and are affected by PD-specific neurodegenerative changes at prodromal stages, the pain modifying effect of l-dopa may be partially mediated through nondopaminergic systems.^9,19,20,44,74^ Accordingly, duloxetine led to some degree of pain relief in an open-label study.^49^ Cannabis has shown an ability to markedly reduce both sensory and affective facets of pain in PwP.^132^ Interestingly, an oxycodone RCT failed to reach significance for the primary end point of reducing 24 hour pain scores.^144^ There was a trend reduction in pain, and the dosage may have been inadequate. However, opioidergic circuitry is known to be perturbed by PD pathophysiology, and this may affect the efficacy of opioid analgesia.^54,115,136,140^ Safinamide, with actions on dopamine through monoamine oxidase-B inhibition as well as modulating abnormal glutamate release, has also shown a benefit in PwP.^26,27,65^ Rotigotine, a purely dopaminergic agonist, produces limited benefit for overall pain in PwP suggesting that safinamide may well impart a benefit through glutamatergic actions and this warrants future investigation.^124^ However, there remains a paucity of robust studies with the Movement Disorder Society non-motor symptoms treatment recommendation identifying only 2 as sufficiently high quality to include.^131^ The multiplicity of neurotransmitter systems through which these drugs act eludes to the complexity of pain in PD. Future research should use refined populations, or those large enough for substratification, to further elucidate how these interventions differentially interact with PD subtypes.

### 3.3. Utility of animal models

Animal models offer a unique opportunity to probe mechanisms of pain and pharmacotherapy. This has been well reviewed for PD,^55,147^ but remains understudied in AD. Mirroring clinical populations most studies report altered pain thresholds compared with controls.^7,59,67,94,99,105,133,137^ A chemically induced model of osteoarthritis through an intra-articular injection of monosodium iodoacetate within transgenic TASTPM AD mice has provided insights into interactions between clinically relevant pain, neurodegenerative pathophysiology, and opioid analgesia.^4,5^ TASTPM mice demonstrate an age-dependent reduction in thermal nociception that coincides with amyloid pathology in pain-related brain regions.^4^ Naloxone, an opioid antagonist, restored thermal nociceptive thresholds to that of wild-type controls. Mice modelling with combined AD and osteoarthritis exhibited impaired mechanical hypersensitivity and a lack of weight asymmetry. Subsequent administration of morphine not only produced an antinociceptive effect but also increased the noxious threshold significantly greater than that seen in wild-type animals.^5^ Conversely, gabapentin showed no efficacy. Thus, altered processing within opioidergic circuitry may partially mediate altered pain processing as well as influence both efficacy and centrally mediated side effects of opioidergic pharmacotherapy. Additional preclinical investigation may yield similar avenues for translational investigation.

## 4. Conclusion

Pain processing is altered in both AD and PD, but research to date has been focussed on evoked pain. During chronic pain, structural and functional reorganisation that takes place can be conceptualised as normal pain processing by the nervous system interacting with a given aetiology to produce a novel chronic pain brain state.^146^ These perturbed states further interact with neurodegenerative pathophysiology in a manner yet to be investigated; whether this produces differential responses to analgesic pharmacotherapy to those seen in the general population remains unclear. However, the theoretical basis outlined here is compelling and mechanistic-level investigation will be crucial to translate our emerging understanding of dysfunctional pain processing to inform safe and effective clinical management. Although our focus here has been on AD and PD, these constructs likely extend to other neurodegenerative diseases that require similar mechanism-based investigation to facilitate therapeutic development.

## Conflict of interest statement

C. Ballard reports grants and personal fees from Acadia pharmaceutical company, grants and personal fees from Lundbeck, personal fees from Roche, personal fees from Otsuka, personal fees from Biogen, personal fees from Eli Lilly, personal fees from Novo Nordisk, personal fees from AARP, grants and personal fees from Synexus, and personal fees from Exciva, all outside the submitted work. The remaining authors have no conflicts of interest to declare.
